# Enhancing collagen based nanoemulgel for effective topical delivery of Aceclofenac and Citronellol oil: Formulation, optimization, in-vitro evaluation, and in-vivo osteoarthritis study with a focus on HMGB-1/RAGE/NF-κB pathway, Klotho, and miR-499a

**DOI:** 10.1007/s13346-024-01548-3

**Published:** 2024-03-19

**Authors:** Reem Abd Elhameed Aldeeb, Sherihan Salaheldin Abdelhamid Ibrahim, Islam Ahmed Khalil, Ghada Mohamed Ragab, Amira Ahmed El-Gazar, Amal Anwar Taha, Doaa Hussien Hassan, Asmaa Ahmed Gomaa, Mona Kamal Younis

**Affiliations:** 1 Department of Pharmaceutics, College of Pharmaceutical Sciences and Drug Manufacturing, Misr University for Science and Technology, 6th of October City, 12566 Egypt; 2 Department of Pharmacology and Therapeutics, Faculty of Pharmacy, Pharos University, Alexandria, Egypt; 3 Department of Pharmacology and Toxicology, College of Pharmaceutical Sciences and Drug Manufacturing, Misr University for Science and Technology, 6th of October City, 12566 Egypt; 4 Department of Pharmacology and Toxicology, Faculty of Pharmacy, October 6 University, 12585 Egypt; 5 Department of Pharmacology and Toxicology, Faculty of Pharmacy, Ahram Canadian University, 6th of October City, 12585 Egypt

**Keywords:** Nanoemulgel, Nanoemulsion, Optimization, Aceclofenac, Citronellol, Osteoarthritis, HMGB-1, Pseudo-ternary phase diagram, D-Limonene, Collagen

## Abstract

The majority of conventional osteoarthritis (OA) treatments are based on molecular adjustment of certain signaling pathways associated with osteoarthritis (OA) pathogenesis, however there is a significant need to search for more effective and safe treatments. This study centers around formulating Aceclofenac (ACF) with high bioavailability in combination with Citronellol oil and collagen. The optimal concentrations of Citronellol oil/D-Limonene oil, Tween 80, and Transcutol HP were determined using a pseudoternary phase diagram. The formulated nanoemulsions were studied for thermophysical stability. Thermodynamically stable formula were analyzed for droplet size, zeta potential, and in-vitro permeation. Then, collagen based nanoemulsion were prepared to capitalize on its efficacy in reducing osteoarthritis side effects and characterized for nano size properties. Formulae F10 and F10C were chosen as optimum nanosize formula. Hense, they were prepared and characterized as nanoemulgel dosage form. The nanoemulgel formulae F10NEG1 and F10CNEG1 showed reasonable viscosity and spreadability, with complete drug release after 4 h. These formulae were chosen for further In vivo anti-OA study. Collagen based ACF/citronellol emugel were able to modulate HMGB-1/RAGE/NF-κB pathway, mitigating the production of inflammatory cytokine TNF-α. They were also able to modulate Klotho and miR-499, reducing serum CTXII and COMP, by reducing the cartilage destruction. Histological investigations validated the efficacy, safety, and superiority of Aceclofenac in combination with Citronellol oil and collagen (F10CNEG1) over solo the treated group (F10NEG1 and blank). Hence, the findings of the current work encourage the use of this promising combined formula in treatment of OA patients.

**Graphical abstract:**

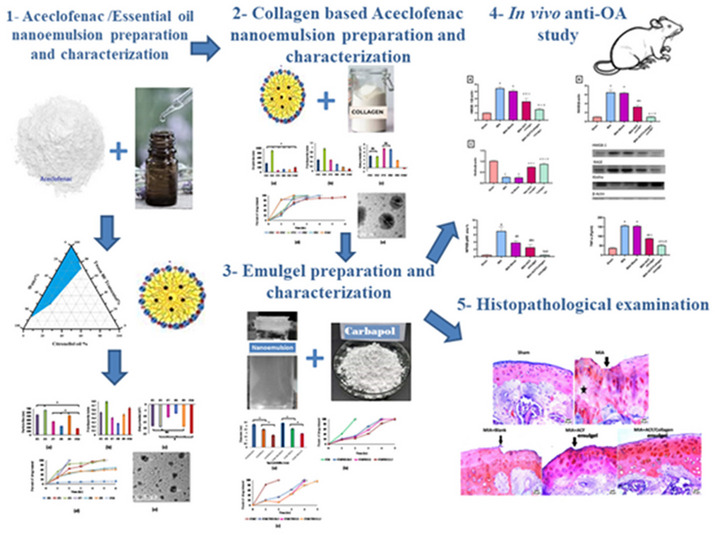

## Introduction

Arthritis is a joint disorder that causes swelling and irritation in the joints, difficulty moving, and severe pain that interferes with a patient's life [1]. Most treatment protocols aimed to adjust certain signaling pathways involved in OA pathogenesis molecularly, however a high need to formulate a convenient formula with high bioavailability and margin of safety. Aceclofenac (ACF) is a nonsteroidal anti-inflammatory drug (NSAID) that relieves pain and inflammation associated with arthritis by inhibiting the secretion of pro-inflammatory substances. ACF is not only used to treat osteoarthritis, but also in rheumatoid arthritis, and spondylitis for its inhibitory potential to cyclo-oxygenase, which participates in the synthesis of prostaglandins that causes pain, swelling, fever, and other inflammatory symptoms [2]. Citronellol oil is a potent terpene essential oil found in citronellol, eucalyptus, catnip, citrus fruits, basil, lavender, black pepper, fennel, lemon, rose geranium, chamomile, and sandalwood. It can help reduce inflammation [3, 4]. The absolute bioavailability of oral ACF is around 15% [5] which is extremely low and is most likely due to its extensive metabolism, and the bioavailability may be lower if the drug is taken in tablet form due to its lower water solubility [6]. Recently, transdermal drug delivery systems have been extensively investigated as an alternative route for delivering NSAIDs. They have the advantage of improving ACF bioavailability as well as reducing gastrointestinal disturbances associated with oral intake of NSAIDs, hence more patient compliance [7]. The increase in bioavailability is most likely related to higher site delivery especially in arthritic conditions, avoidance of hepatic first-pass metabolism, lack of drug-drug interaction, prolonged drug release, and controlled therapeutic responses [7, 8]. Several methods for increasing drug penetration through the skin have been studied. Among these are penetration enhancers, such as natural terpenes, which are a safe and effective class of penetration enhancers [9–11]. Terpenes such as D-limonene and citronellol have been shown in experimental studies to be safe on the skin and effective in improving drug delivery across the skin [12, 13]. Collagen is a dietary supplement made from animal or fish materials [14]. It is rich in amino acids that play a major role in building joint cartilage and may have anti-inflammatory effects [15, 16]. Recent studies showed that collagen is effective in improving osteoarthritis symptoms [16–18]. Emulgel is a new technique for dual control of emulsion and gel release in which the emulsion's stability increases when combined with a gel [19, 20]. Collectively, ACF nanoemulgel with terpene oil was prepared to improve its permeation through the skin. An additional benefit was suspected with the incorporation of collagen, providing a more efficient antiarthritic effect. Furthermore, this study tracked the usefulness of the optimized formulae to attenuate mono-iodoacetate (MIA)-induced osteoarthritis in rats via modulating HMGB-1/RAGE/NF-κB pathway, Klotho, and miRNA-499a that are implicated in the pathogenesis of OA.

## Materials and methods

### Materials

ACF was kindly provided by Bristol Mayers Squibb (Cairo, Egypt). Citronellol, D-Limonene, Tween 80 (polyoxyethylene (20) sorbitan monooleate) and Transcutol HP (diethylene glycol monoethyl ether), Collagen ex. Marine Fish were purchased from Sisco research laboratories (Mumbai, India). Carbopol 942 and triethanolamine were purchased from Alpha chemika company (Mumbai, India). For osteoarthritis induction mono-iodoacetate (MIA) was purchased from Sigma-Aldrich (Missouri, USA).

### Methods

#### Drug solubility test

The solubility of ACF in Oil (Citronella and D-Limonene), Tween 80 and Transcutol HP was estimated by adding excess amount of the drug in 2 mL of each of the excipients and mixing them in a vortex mixer for 5 min. Then the mixture was immersed in an isothermal water bath at 37 ± 1 °C for 48 h. It was then centrifuged (Microcentrifuge, Shanghai Surgical Instrument Factory, model 800) at 3000 rpm for 15 min, and separated through a 0.45 μm membrane filter. The drug content was determined using UV-Vis. spectrophotometer (Shimadzu, U-1800 spectrophotometer, Tokyo, Japan) at λmax 276 nm. All the experiments were done in triplicate [21].

#### Pseudo-ternary phase diagram and nanoemulsion (NE) preparation

Aqueous phase titration was used to figure out the best ratio of oils (D-Limonene, Citronellol), surfactants (Tween 80), and co-surfactants (Transcutol HP). Surfactant/cosurfactant proportions from 1:1 to 1:9 were combined with oil in ratios from 1:9 to 9:1. The end point of titration is the first turbidity appearance. The results were plotted on the Grapher program (Version 8.1.388) to detect the emulsification regions. Regarding this study, 12 formulas from (F1-F12) were selected from high emulsification region on pseudo-ternary phase diagram which allows for increased versatility in identifying the optimal dosage composition [22, 23]. ACF 30 mg was dissolved in 1 g of NE containing oil (citronellol and D-Limonene), surfactant (Tween 80), and cosurfactant (Transcutol HP) that was weighed into glass vials. The combination was gently stirred and sonicated (Ultrasonic Lc 60 H Sonicator, Elma, Germany) until ACF was completely dissolved. The mixture was kept at room temperature until it was utilized [24].

#### Evaluation of the prepared NE

##### Physical stability

The prepared NE were centrifuged at 13,000 rpm for 30 min at 4 °C and 25 °C. Six cycles of centrifugation were performed with 48 h. storage at each temperature. Stability of test formulas was decided by examining their appearance for turbidity, phase separation, precipitation, and creaming [25].

##### Drug content estimation

ACF in Table 1 was extracted from each drug formulae using methanol and sonicated for 5 min. The methanolic extract was analyzed for ACF content spectrophotometrically at a wavelength of λmax 276 nm [26].

**Table 1:** Ingredients of prepared NEs

**Code**	**Drug** **g**	**Limonene oil** **g**	**Citronella oil** **g**	**Tween 80** **g**	**Transcutol** **g**	**Water** **mL**
F1	0.3	1		2.5	2.5	Complete volume to 100 mL
F2	0.3	1		3.5	1.5	
F3	0.3	1		1.5	3.5	
F4	0.3	2		2.5	2.5	
F5	0.3	3		3.5	1.5	
F6	0.3	4		1.5	3.5	
F7	0.3		1	4.5	1	
F8	0.3		1	6.5	3	
F9	0.3		1	8.5	1	
F10	0.3		1	4.5	4.5	
F11	0.3		3	4.5	3	
F12	0.3		4	4.5	1	

##### Droplet size and zeta potential measurement

The droplet size distribution and surface charge of the prepared NE were measured using dynamic light scattering (DLS) method using Zetasizer 2000 (Malvern Instruments, Malvern, UK) with backscatter detection at 173 °C; refractive index 1.330. The formulations were diluted with distilled water. Samples were placed into a disposable zeta cell and zeta potential values were measured at a temperature of 25 °C at a scattering angle of 90° [27].

##### In vitro permeation studies

One mL of NE was placed in dialysis bags and suspended in 50 mL of phosphate buffer pH 7.4 at 37 °C and 100 rpm with a USP dissolution test; Paddle type apparatus (Dr. Schleuniger Pharmatron, type Diss 6000, Solothurn, Switzerland). Three mL of the dissolution medium were withdrawn and replaced with fresh medium at intervals of 1, 2, 3, 4, 5, and 6 h. The amount of ACF released from NE was determined spectrophotometrically at λmax 276 nm [28].

##### Morphological study

The morphological analysis of optimized NE formula was conducted using transmission electron microscopy (TEM) (Joel JEM 1230, Tokyo, Japan). This involved preparing the sample by drying it onto a carbon-coated grid, followed by negative staining using phosphotungstic acid in an aqueous solution. Once the phosphotungstic acid had dried, the sample was then examined under TEM for detailed observation operated at 60–80 kV at 1550 × magnification [29].

#### Preparation and evaluation of collagen-based nanoemulsion (CNE)

Collagen solution was prepared by dissolving 10 mg collagen in 3.3 mL of 5% acetic acid. The collagen solution was then mixed with 2 mL of selected formulae F2, F3, F7, F8, F9 and F10 to form F2C, F3C, F7C, F8C, F9C and F10C collagen based NE [30].The collagen based NE formulas were assessed for ACF content, droplet size and surface charge and in vitro permeation studies in the same steps as that previously mentioned for nanoemulsion [31]

#### Preparation of nanoemulgel (NEG) and collagen-based nanoemulgel (CNEG)

Nanemulsion (F10) and Collagen based nanoemulsion (F10C) were selected as the optimized formulae to be incorporated into the Carbopol gel. The gel bases were prepared in three different concentrations (0.5%, 1% and 1.5% w/w) by adding a certain amount of Carbopol 942 in distilled water with constant stirring at 2000 rpm using a magnetic stirrer for 60 min. After which F10 and F10C were blended in the three different gel concentrations in a ratio of (1:1) to prepare the nanoemulgel formulae F10NEG0.5, F10CNEG0.5, F10NEG1, F10CNEG1, F10NEG1.5 and F10CNEG1.5, respectively. Finally, the pH was adjusted to 6.5 by adding triethanolamine [32].

#### Characterization of prepared nanoemulgel

##### Drug content estimation

500 mg of prepared nanoemulgel formulae was dissolved in 50 mL of phosphate buffer pH 7.4. The solution was filtered through filter paper and the drug content was measured spectrophotometry at λmax 276 nm [33].

##### Homogeneity

All prepared nanoemulgel formulae were evaluated for homogeneity by visual inspection after placing the gels in the container. They are examined for their appearance and the presence of any aggregates [34].

##### Rheology study

The prepared nanoemulgel formulae were estimated using a Brookfield viscometer (Cone/plate Brookfield, USA). Using spindle No. 40, 0.5 g of the formulation was applied to the plate and rotated from 5 to 240 rpm over a wide range of shearing rates (from 22.5 to 1800 s^-1^). At each speed, the corresponding contact reading was observed [35]. The degree of pseudoplasticity (Farrow's constant) and viscosity were determined. To study the flow behavior of the gels, Farrow's equation was applied as in Eq. 1 [36]:

1 $$\text{Log G }=\text{ N LOG F}-\text{Log }\eta$$

where G is the shear rate (s^−1^), N is Farrow's constant, F is shearing stress (dyne/cm2) and $\eta$ is viscosity (cP). Log G was plotted against Log F to obtain the value of N, which indicates deviation from Newtonian flow.

##### Spreadability

A 0.5 g of the prepared nanoemulgel formulae was spread on a circle with a diameter of 2 cm previously marked on a glass plate and then a second glass plate was employed. After adding 100 g weight on the top glass plate for 5 min, the diameter of the spread gel circle was measured [37].

##### In vitro permeation studies

The release of ACF from nanoemulgel preparations was studied. 1 g sample was placed in dialysis bags and suspended in 50 mL of phosphate buffer (pH 7.4) at 37 °C under stirring at 100 rpm using dissolution apparatus (USP rotating paddle dissolution test apparatus; Dr. Schleuniger Pharmatron, type Diss 6000, Switzerland). Three mL of the dissolution medium was withdrawn and replaced with fresh medium at different intervals of 1, 2, 3, 4, 5 and 6 h. The amount of drug released was measured by spectrophotometry at λmax 276 nm [28].

#### In vivo animal studies

##### Animal handling

The ethical committee for animal experiments at Collage of Pharmaceutical Sciences and drug Manufacturing, Misr University for Science and Technology, approved the protocol of the in vivo study (approval no. PH 19). The study also followed the guidelines for the handling and use of laboratory animals published by the US National Institutes of Health (8th edition, NIH Publication, 2011) [38].

##### Study design and osteoarthritis induction

Rats were randomly allocated into five groups (10 rats/group). The first one was the sham control group injected with 50 μL of physiologic saline into their right knees. The rest of animal’s groups (n = 40) received 3 mg monosodium iodoacetate (MIA) that was dissolved in 50 μL of sterile saline by unilateral injection into the right knee joint prior to the knee shaving process as described previously [39]. The rats received MIA were distributed randomly as follow: the first set was signified as MIA and left without any further intervention and served as a positive control; the second set signified as blank and they were treated with nanoemulgel Citronellol topically (40 mg/day); the third set were treated with nanoemulgel ACF (12 mg/day); the fourth one: OA rats received ACF/collagen combination at a dose of (12 mg ACF and 7.5 mg collagen). All the treatments were given for 14 consecutive days (4 times/day).

Many advanced computerized methods are available for randomization; however a simple randomization method was applied with some precautions to decrease interference and bias. First, the purchasing of animals was done at the National Research Centre (NRC, Giza, Egypt), which is a place known for supplying animals with high-quality health care. After that, all the animals were examined to ensure their sex, weight, and health state, and any animal that did not meet the specifications or inclusion criteria was excluded. Then a simple randomization method was applied by pooling all the animals in one large cage, then a blind allocation was done with the aid of a lap technician into different groups, and a label with symbols was added to each cage. Then animals were left one week to adapt to the vivarium (automatically controlled temperature, humidity, ventilation, and 12-h light/dark cycle) with limited access to food and water. On day one of the experiment, the lap technician removed the old labels, and the final group number was prepared by one of the team researchers and then added by the lap technician sequentially to each cage without knowing what this number refers to. Besides, the different analyses and measurements (histological and biochemical) were done blindly, and the key was available only to the researcher with a formal analysis task.

##### Serum parameters

Twenty-four hours after the last treatment, blood samples from each animal were collected through the retro-orbital sinus while they were all anaesthetized with pentobarbital sodium (200 mg/kg, IP). Serum was then used to assess TNF-α, CTX-II and COMP using enzyme-linked immunosorbent assay (ELISA) kit (MyBioSource, CA, USA) according to the manufacturer’s instructions [40].

##### Tissue parameters

Scarification by cervical dislocation was done immediately after blood collection, then knee tissue was excised, rinsed with ice-cold saline, and separated into two sets. The first set was used for the different biochemical analysis (n = 6) while the second one (n = 3) was preserved in 10% formalin for histological and immunohistology investigation [41].

##### Western blot

Klotho, HMGB-1, RAGE protein expression were determined by western blotting [42]; the primary antibody was purchased from Thermo Scientific Co., (IL, USA) in accordance with the previously reported procedure [43]. The values' optical density (OD) was normalized against beta-actin.

The band intensity was analysed by the ChemiDocTM imaging system with Image LabTM software version 5.1 (Bio-Rad Laboratories Inc., Hercules, CA, USA). The results were expressed as arbitrary units after normalization for β-actin protein expression, and then multiple comparisons were performed using one-way analysis of variance (ANOVA) among groups, followed by Tukey’s multiple comparison test to compare the mean between each group. The values obtained were then expressed as mean ± S.D. and analyzed using GraphPad Prism 8.0 [44].

##### Quantitative real-time PCR for miR-499-5p gene expression

RNA was extracted from knee joint tissues using the miReasy mini kit and methodology for purification of serum total RNA, which involved miR and long noncoding RNA (Qiagen, Valencia, CA, USA). The purity of RNA samples was measured and evaluated using the NanoDrop^®^ (ND)-1000 spectrophotometer (NanoDrop Technologies, Inc. Wilmington, NC, USA). Reverse transcription and quantitative real-time PCR (qPCR) were utilized to find the mature miR-499-5pprimer. Twenty uL of total RNA were reverse transcribed using the miScript II RT kit from Qiagen in Valencia, California, the United States. Using the miScript SYBR^®^ Green PCR kit and procedure for mature miRNAs quantitative assessment (Qiagen, Valencia, CA, USA) and the specificmiR-499-5p primer, quantitative RT-PCR was carried out in a total volume of 25 L per reaction volume [45]. The primer sequences for the qPCR were: miR-499-5p moves ahead with 5′-TTAAGACTTGCAGTGATGTTT-3 and reverses with 5′-GTGCAGGGTCCGAGGT-3′; and β-actin moves ahead with 5′-CGTTGACATCCGTAAAGACCTC-3′ and reverses with 5′-TAGGAGCCAGGGCAGTAATCT-3′. By using Equation 2 − ∆∆Ct, the fold change in miRs expression was estimated.

#### Histopathological examination

##### Hematoxylin and eosin & safranin O-fast solution staining

Specimens were fixed in 10% neutral buffer formalin, then decalcified by EDTA 10% solution, trimmed, washed in water, dehydrated in ascending grades of ethyl alcohol, cleared in xylene, and embedded in paraffin. Thin section (4-6µ) was processed and stained with Hematoxylin & Eosin stain and safranin O stain [46].

##### Immunohistochemical staining protocol

Paraffin sections were mounted on positively charged slides by using avidin biotin- peroxidase complex (ABC) method [47]. Rabbit NF-κB-p65 polyclonal Antibody (Elabscience, Cat# E-AB-32232, Dil.: 1:100). Sections from each group were incubated with these antibodies, then the reagents required for ABC method were added (Vectastain ABC-HRP kit, Vector laboratories). Marker expression was labeled with peroxidase and colored with diaminobenzidine (DAB, produced by Sigma) to detect antigen-antibody complex. Negative controls were included using non-immune serum in place of the primary or secondary antibodies. IHC stained sections were examined via using Olympus microscope (BX-53).

#### Statistical analysis

Values are expressed as mean ± S.D. and were analyzed using the GraphPad Prism 8.0 (GraphPad Prism Inc., La Jolla, CA, USA). Multiple comparisons were performed using One-way analysis of variance (ANOVA) was used among groups followed by Tukey’s multiple comparison test to compare the mean between each group. A p-value < 0.05 was considered significant (*) statistically.

## Result and discussion

The OA is one of the most common diseases that can restrict the patient’s movement and have a debilitating effect causing major subchondral, cartilage and synovial damage leading to whole arthropathy [48]. Knee OA is the most prevalent type of OA, which is considered the 4th leading cause of disability worldwide with no radical cure [49]. Moreover, despite its great incidence, prevalence and detrimental effects on people's quality of life related to their health, it is commonly disregarded in both international and national strategic plans for the management of chronic diseases [50]. The existing socioeconomic effects of OA are unsustainable due to the rising costs of healthcare and joint replacement, which is a significant global concern [51, 52].

The existing osteoarthritis pharmaceutical therapies have no discernible disease-modifying impact. The ACF is a NSAID that possess anti-inflammtory effect in management of osteoarthritis. However, it has a number of drawbacks, including poor solubility, low bioavailability, and gastrointestinal adverse effects when used over an extended period of time [53]. It was warranted to distribute ACF using drug delivery nanotechnology to improve its safety profile. Nanoemulgel is a novel drug delivery system that combines the benefits of nanoemulsions and hydrogels for improved drug solubility and sustained release. It’s widely used in pharmaceuticals, particularly for topical and transdermal drug delivery, due to its enhanced permeation and retention properties. Recent studies showed promising results, including the delivery of anti-inflammatory, anti-fungal, and anti-acner agents [54]. In the current study we assessed the invitro nanodrug delivery and the invivo anti-inflammatory effect with the suggested molecoular effects of ACF emugel and collagen incorporated nanoemulsion ACF based gel in ameliorating MIA induced OA in rats.

### Drug solubility test

ACF is a poorly water-soluble drug belonging to BCS class-II. The solubility of ACF in water was 0.071 mg/ml. The solubility of ACF in citronellol oil and D-Limonene oil was found to be 5.26 ± 0.7 mg/ml and 5.18 ± 0.2 mg/ml, respectively while the solubility of ACF in Tween 80 and Transcutol HP was 62.6 ± 0.13 mg/ml and 300.2 ± 0.05 mg/ml, respectively. There was a significantly high ACF solubility in oil, surfactant and cosurfactant tested media compared to solubility in water (P value < 0.05) [55].

### Pseudo-ternary phase diagram and Nanoemulsion (NE) preparation

**Fig.1:**
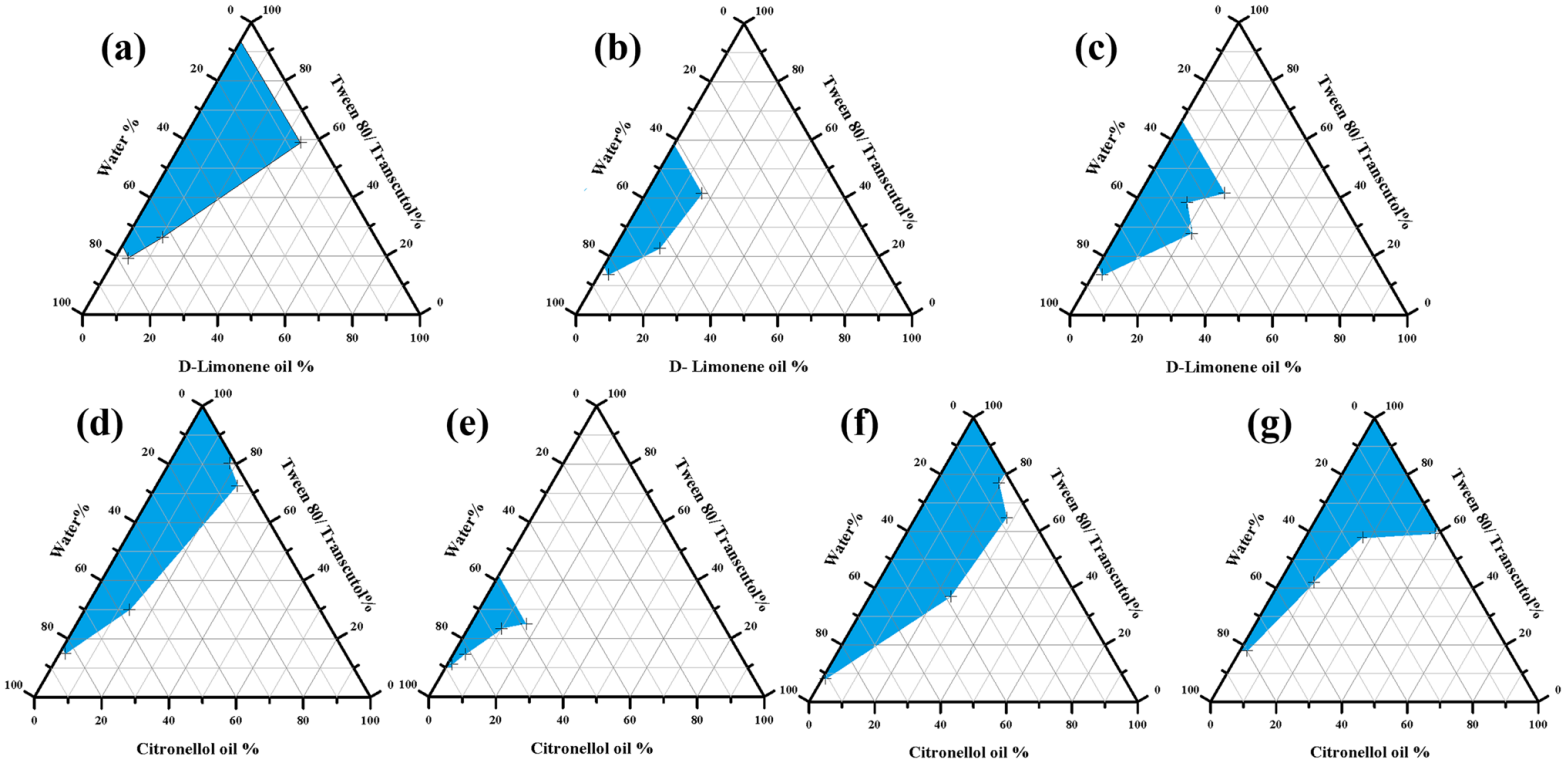
Pseudo-ternary phase diagrams of systems showing high emulsification region composed of oil (Limonene /Citronellol), Tween 80 and Transcutol mixture (Smix), and Water with Smix ratios: **a** 1:1 with Limonene oil **b** 2:1 with Limonene oil **c** 1:2 with Limonene oil **d** 1:1 with Citronellol oil **e** 2:1 with Citronellol oil **f** 4:1 with Citronellol oil **g** 8:1 with Citronellol oil

Grapher software version (8.1.388) illustrated the result of aqueous phase titration experiment (Fig. 1). High surfactant/co-surfactant (Smix) was sufficient to lower interfacial tension between oil and water and prevent oil droplets aggregation to produce clear NE [56]. F1-F12 NE were titrated with the maximum amount of water in the water titration experiment, this was confirmed from diagram obtained by the Grapher. These formulas were prepared (Table 1) and evaluated for thermodynamic physical stability.

### Evaluation of the prepared NE

#### Physical stability

It was observed that F2, F3, F7, F8, F9, and F10 showed up clear and thermodynamic stable systems, while other systems showed phase separation after centrifugation test. These indicates that high concentrations of the surfactant and co-surfactant compared to oil in the system increased the nanoemulsion system entropy, lowering the oil/water interfacial tension and decreasing its free energy so maintained the thermodynamic stability of these formulas. In addition, the high amount of non-ionic surfactant may create a steric stabilization that provides a rigid film surrounding the oil droplets and prevents self-coalescence. Along these lines, F2, F3, F7, F8, F9, and F10 were consequently decided to be further assessed in this study [56].

#### Drug content

The drug contents of the selected NEs F2, F3, F7, F8, F9 and F10 were 97.63% ± 0.09, 96.92% ± 0.1, 96.90% ± 0.11, 97.23% ± 0.07, 96.43% ± 0.1 and 97.74% ± 0.14, respectively, that indicated uniform distribution of the drug.

#### Droplet size and zeta potential analysis

**Fig. 2:**
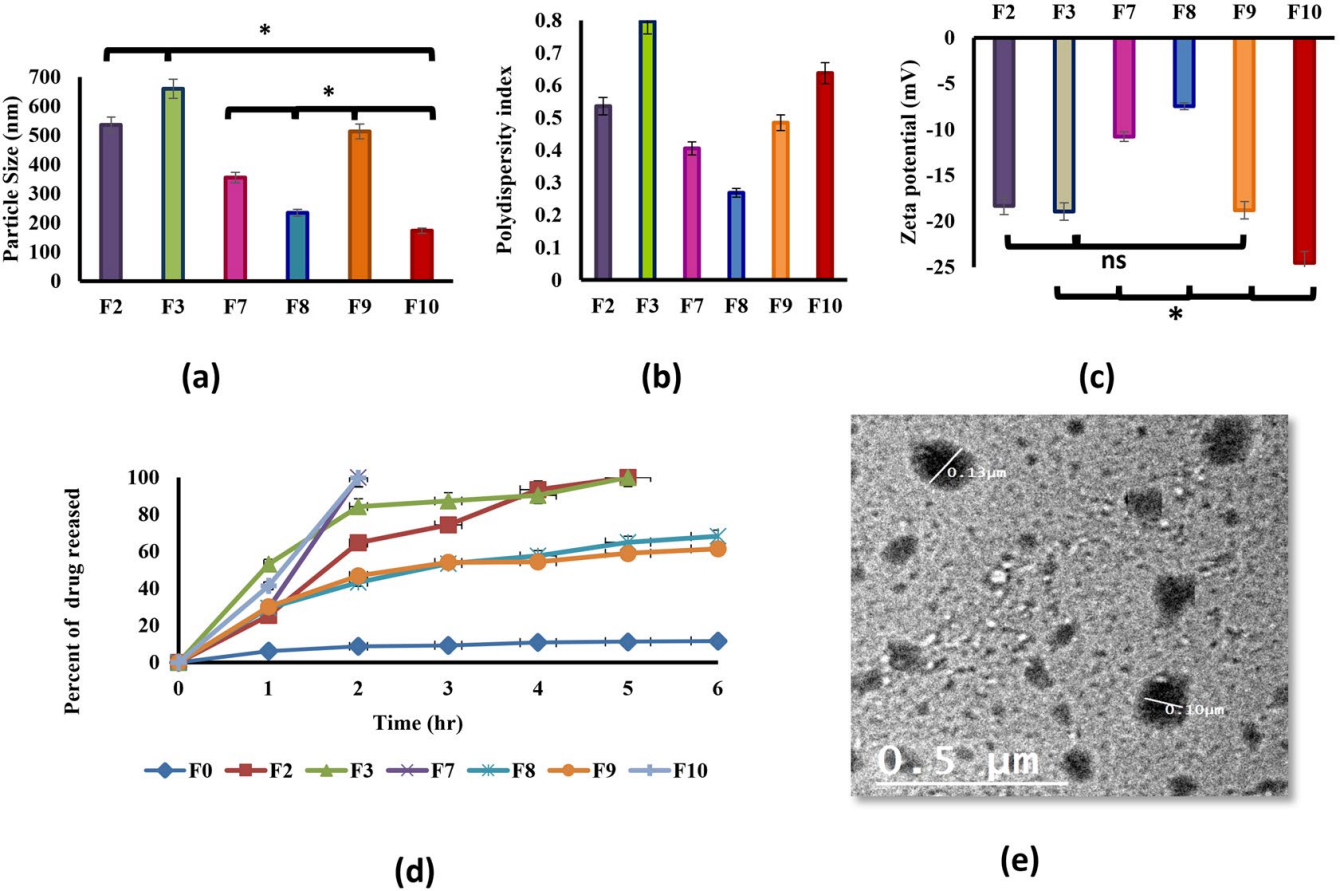
Characterization of prepared nanoemulsion formulae **a** average droplet size, **b** polydispersity index (PDI), **c** average zeta-potential, **d** In-vitro permeation release, **e** transmission electron microscope image of nanoemulsion formula (F10)

The NE droplet size controls the rate and extent of drug release and drug absorption [57]. The droplets size of the selected prepared NE formulae were found to be 536.4 ± 28, 660.5 ± 37, 355.3 ± 41, 233.7 ± 31, 513.1 ± 46 and 172.4 ± 16 nm for F2, F3, F7, F8, F9 and F10, respectively. All measured droplet sizes were in the nanosized range since they ranged between 172.4 ± 16 nm to 660.5 ± 37 nm with PDI (polydispersity index) less than 1 which demonstrates the homogeneity of prepared formula (Fig. 2). The measured nanosized of NE may resulted from the presence of relatively high system concentrations of the surfactant and co-surfactant which led to lowering the oil/water interfacial tension [58].

The measured zeta potential ranged between -7.486 ± 0.4 mV to -24.55 ± 2.8 mV. However, the presence of nonionic surfactant Tween 80 decreases the formulae charge but the polyethylene oxide (PEO) groups, (the hydrophilic part of Tween 80), provide steric stabilization (Fig. 2) [59].

#### In- vitro permeation studies

The complete drug release (100%) for prepared F7 and F10 occurred after 2 h and after 5 h for F2 and F3, while the prepared NE formulae F8 and F9 showed 68 and 61% drug release after 6 h, respectively. These results matched well with the results of particle size analysis as F7 and F10 showed smaller particle size (355.3 ± 41 and 172.4 ± 16, respectively) and faster drug release than F2 and F3(536.4 ± 28, 660.5 ± 37, respectively) as small-sized droplets showed greater surface area providing better drug partitioning and release [57]. On the other hand, formulae F8 and F9 had nanoscale particle size and did not completely release after six hours. This could be attributed to their higher surfactant content which might lead to the formation of a thicker interfacial film around the oil droplets that hindered the drug diffusion (Fig. 2) [60]. High surfactant content may also lead to lower drug release, due to the increased viscosity and reduced permeability of the nanoemulsion [61].

#### Morphological study

F10 showed to be the optimum formula since it had the smallest particle size (172.416 nm), highest zeta potential (-24.55 mV), and 100% drug release with the fastest rate (within 2 h). Therefore, it was selected to undergo further studies. The TEM image of F10 NE obviated spherical NE globules less than 200 nm in size that confirmed the results obtained by the droplet size estimation. Spherical and separated globules in the image deduce the system stability and uniformity of oil droplets dispersion in the aqueous phase. Smaller and spherical droplets have lower interfacial tension and higher surface area, which can enhance the stability and performance of the nanoemulsion. Separated globules mean that there is no aggregation or coalescence of the droplets, which can lead to phase separation and instability of the nanoemulsion [62]. A thicker darker wall of NE globules can be seen in the image, which may be ascribed to surface accumulation of ACF (Fig. 2).

### Preparation and evaluation of collagen-based NE

Collagen solution was prepared and added to NE F2, F3, F7, F8, F9 and F10 to form collagen based nanoemulsion F2C, F3C, F7C, F8C, F9C and F10C, respectively. This was done to capitalize on its efficacy in alleviating osteoarthritis symptoms [17, 18] and to reduce the size of collagen particles in the NE to improve its absorption.

#### Determination of drug content

The drug contents of the collagen based nanoemulsion F2C, F3C, F7C, F8C, F9C and F10C were 95.12% ± 0.18, 96.00% ± 0.06, 94.72% ± 0.23, 96.25% ± 0.16, 95.88% ± 0.04 and 96.15% ± 0.09, respectively.

#### Droplet size and zeta potential analysis

**Fig. 3:**
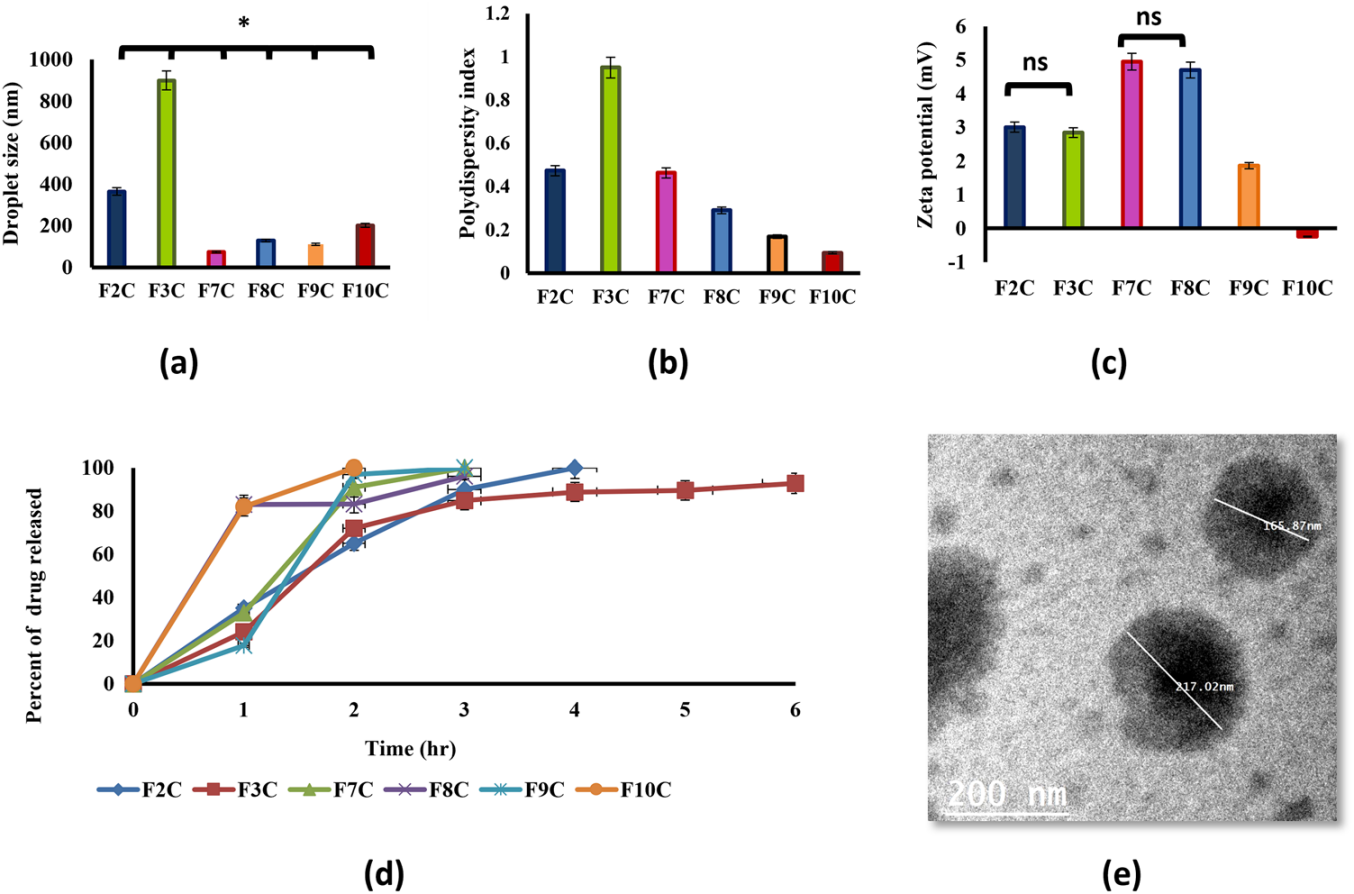
Characterization of prepared collagen based nanoemulsion formulae **a** average droplet size, **b** polydispersity index (PDI), **c** average zeta-potential, **d** In-vitro permeation release, **e** transmission electron microscope image of collagen based nanoemulsion formula (F10C)

Formulae had particle sizes ranging from 73.55 ± 1.15 nm to 899.3 ± 6.53 nm. The PDI values of the formulae were less than one, showing homogeneous preparations, and the zeta potential ranged from—0.25 ± 0.061 to 4.947 ± 0.261, showing that the addition of collagen solution decreased the zeta potential. Yan et al. [63] reported that collagen forms a mesh-like structure around particles, preventing coalescence and increasing particle stability (Fig. 3) .

#### In-vitro permeation studies

The In-vitro permeation study (Fig. 2d) showed complete drug release for all preparation before 4 h hours except F3C which released 92% of drug after 6 h. This could be due to its larger droplet size (899.3 ± 6.53) compared to other prepared formulae (Fig. 3) [57].

#### Morphological study

The TEM studies of F10C (with collagen, Fig. 3e) were done to be compared with previous showed TEM image of F10 (without collagen, Fig. 2e). It was obvious that collagen formed a dense structure around NE droplets preventing droplet accumulation in the preparation (Fig. 3).

### Preparation and characterization of nanoemulgel (NEG)

Optimum Formulae, F10 and F10C were chosen to prepare the nanoemulgel dosage form. F10 and F10C were mixed in a 1:1 ratio with 3 different gel concentrations, 0.5%, 1%, and 1.5% w/w, to prepare the nanoemulgel (NE) formulae F10NEG0.5, F10CNEG0.5, F10NEG1, F10CNEG1, F10CNEG1.5, and F10CNEG1.5, respectively.

#### Determination of drug contents

The drug contents of the F10NEG0.5, F10CNEG0.5, F10NEG1, F10CNEG1, F10NEG1.5 and F10CNEG1.5 were 94.5 ± 0.23%, 95.4 ± 0.28%, 95.11 ± 0.15%, 95.21 ± 0.32%, 93.32 ± 0.19% and 94.42 ± 0.29%, respectively. This result was within the limit (90–110%) according to US Pharmacopeia [64].

#### Homogeneity

All prepared nanoemulgel formulae were homogenous in appearance with no aggregations.

#### Rheology study

**Fig. 4:**
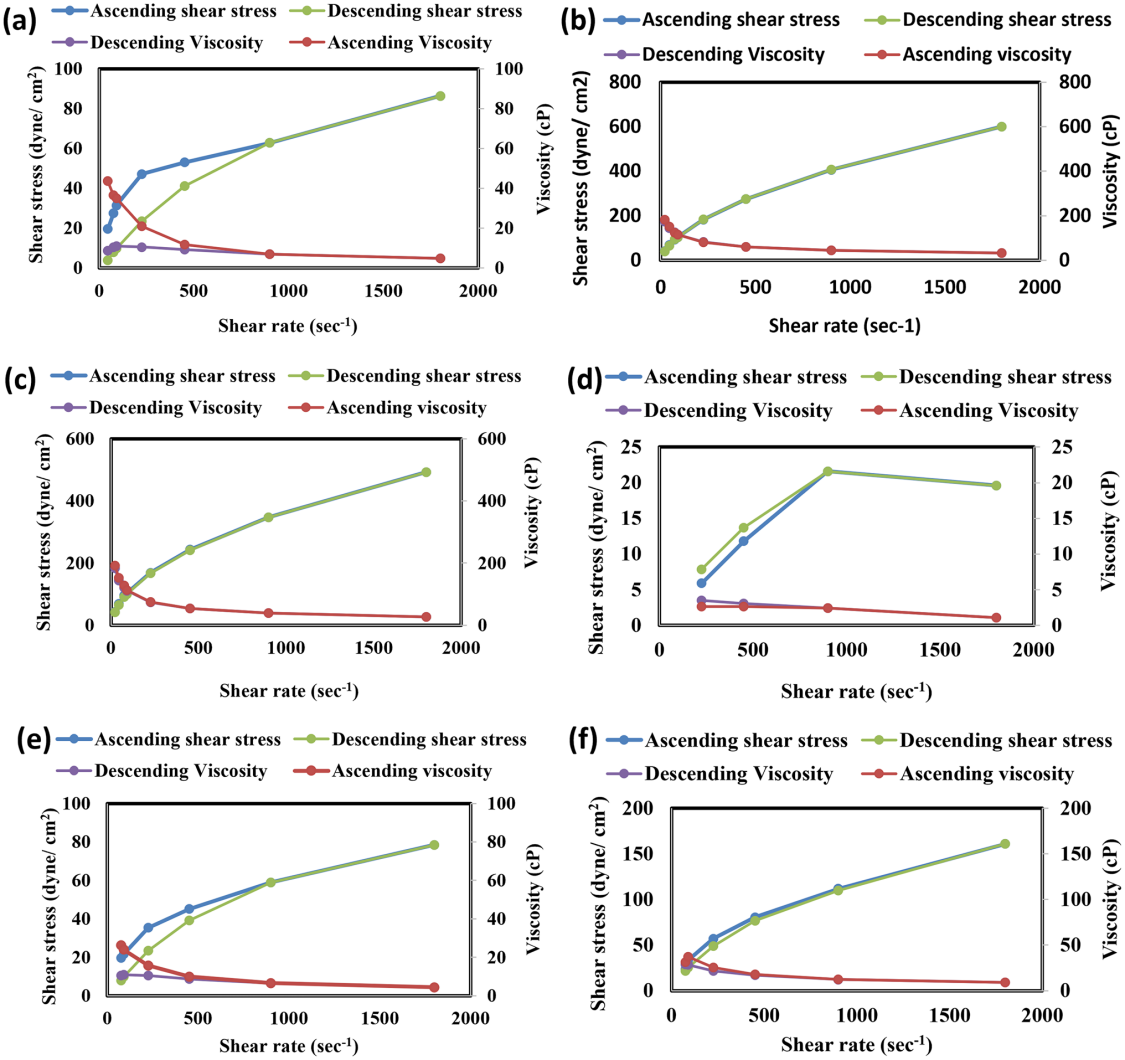
Shear rate against shear stress and viscosity curves for the nanoemulgel formulae **a** F10NEG0.5, **b** F10NEG1, **c** F10NEG1.5, **d** F10CNEG0.5, **e** F10CNEG1 and **f** F10CNEG1.5

The prepared nanoemulgel formulae were placed in a Brookfield viscometer using spindle 40 at 25 °C.An ascending and descending continuous shear was applied ranged from 6 to 240 rpm (Fig. 4). The rheograms revealed that prepared nanoemulgel showed pseudoplastic flow behaviour that is suitable for spreadability on skin surface [65]. The viscosity of F10NEG0.5, F10CNEG0.5, F10NEG1, F10CNEG1, F10NEG1.5 and F10CNEG1.5 were 43.6 ± 12, 3.49 ± 0.12, 183.1 ± 32.9, 26.2 ± 0.05, 191.8 ± 13.5 and 31.4 ± 0.12cp, respectively, at 6 rpm (22.5 s^-1^ shear rate) and 4.8 ± 0.11, 1.09 ± 0.03, 33.4 ± 0.14, 4.36 ± 0.18, 27.4 ± 0.16 and 8.94 ± 0.2cp, respectively, at 240 rpm (1800 s^-1^ shear rate), respectively. It was obvious that increasing the percent of carbopol 942 increased the viscosity of the nanoemulgel formulae. It was observed that formulae containing collagen had lower viscosity compared to other formulae. Sionkowska et al. and Yulong et al. reported that collagen solution is characterized by the typical shear-thinning behaviour reflecting on the decrease in viscosity as the shear rate increases due to the progressive orientation and disentanglement of the collagen polymer chains in acetic acid solution [66, 67].

Farrow’s equation was used to determine farrow's constant (N), which was 26.29, 74, 3.1, 28.33, 3.8 and 12.55 for F10NEG0.5, F10CNEG0.5, F10NEG1, F10CNEG1, F10NEG1.5 and F10CNEG1.5, respectively. Farrow's constant with a value greater than 1 indicates a non-Newtonian behavior (pseudoplastic flow) and a shear rate thinning behavior, in which viscosity decreases with increasing shear stress.

#### Spreadability

**Fig. 5:**
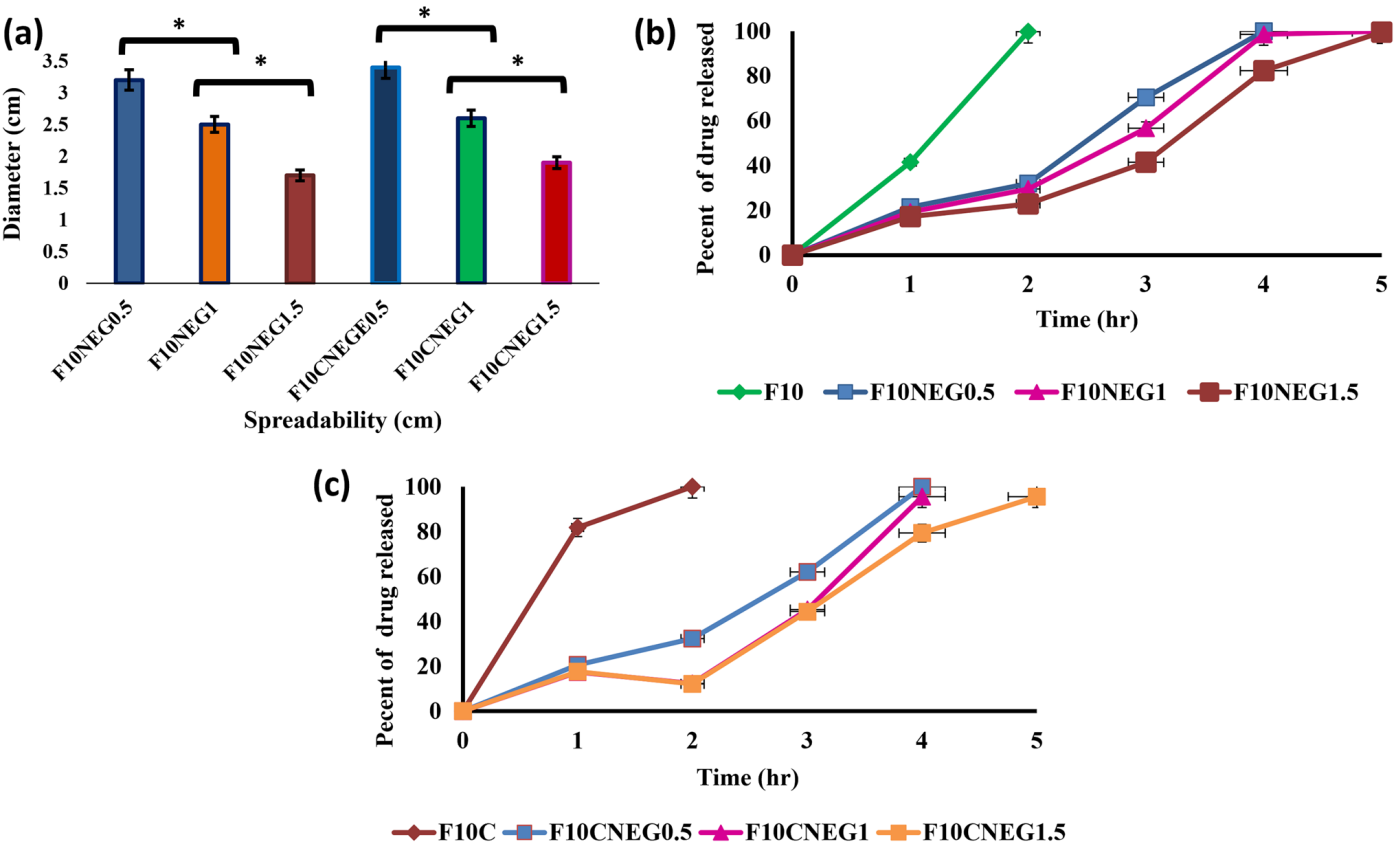
Characterization of prepared nanoemulgel formulae **a** Diameter of spreadability of prepared nanoemulgel formulae after applying 100 g weight, **b** In-vitro permeation release of prepared nanoemulgel formulae and **c** In-vitro permeation release of prepared collagen based nanoemulgel formulae

Spreadability of the formula aids in the uniform utilization of the gel to the skin, which is basic for patient compliance. As a result, spreadability tests for prepared nanoemulgel formulae were directed Spreadability test results (Fig. 5) revealed diameters of 3.2 ± 0.032, 3.4 ± 0.022, 2.5 ± 0.017, 2.6 ± 0.014,1.7 ± 0.012, and 1.9 ± 0.023 for F10NEG0.5, F10CNEG0.5, F10NEG1, F10CNEG1, F10NEG1.5, and F10CNEG1.5, respectively. These values were regarded as reasonable formulae spreadability.

#### In-vitro permeation studies

The results of in vitro release permeation (Fig. 5) showed that complete release of collagen-containing formulae was faster than that of other prepared formulae due to their lower viscosity. Formulae with lower Carbopol 942 contents also had a faster complete release due to their lower viscosity. The viscosity of the formulation was important in controlling drug permeation as high viscosity may result in a more rigid structure with a lower drug release rate [68].

The nanoemulgel formulae F10NEG1 and F10CNEG1 demonstrated reasonable viscosity and spreadability, with complete drug release after 4 h. These formulae were chosen for further In-vivo study.

#### In vivo studies

##### Impact of ACF nanoemulgel alone (F10NEG1) or in combination with collagen (F10CNEG1) on serum CTXII and COMP in MIA-provoked osteoarthritis

**Fig. 6:**
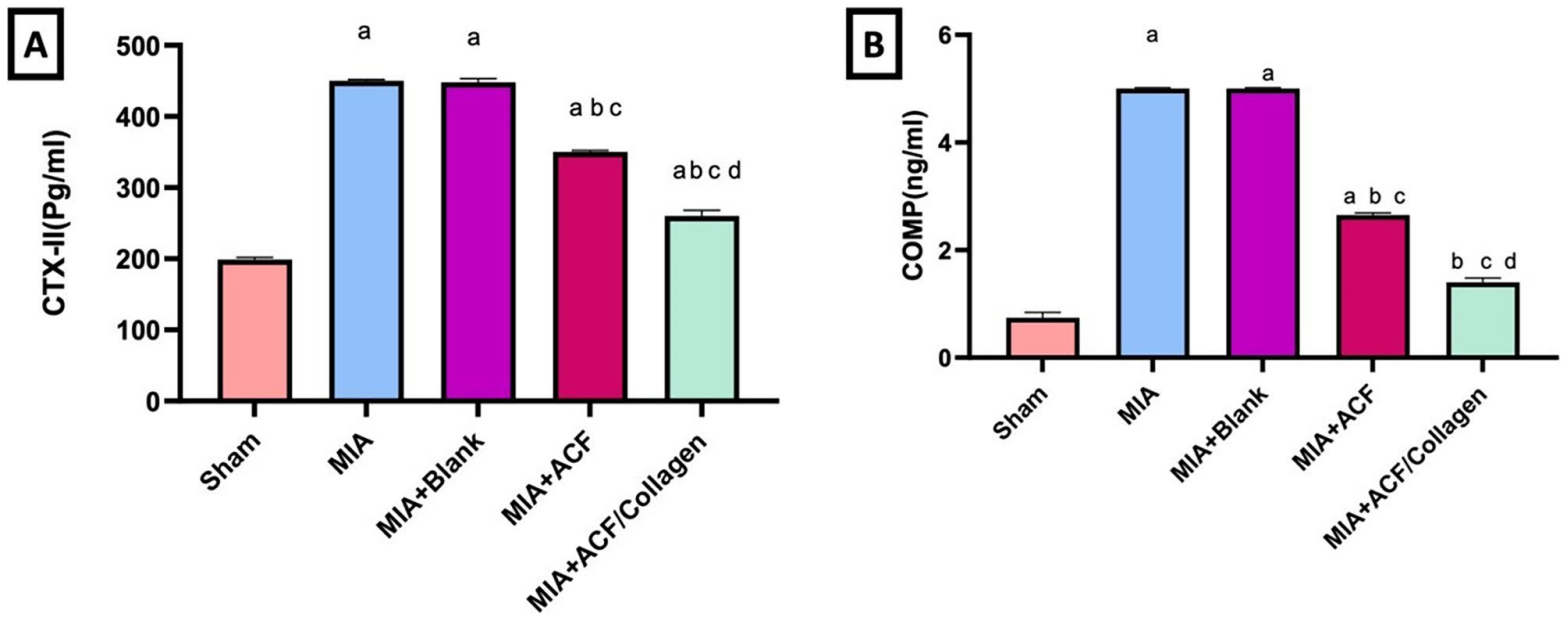
Impact of ACF nanoemulgel alone (F10NEG1) or in combination with collagen (F10CNEG1) on serum **A** CTXII and **B** COMP in MIA-provoked osteoarthritis. Values are presented as mean (n = 6) ± SD and statistical analysis was carried out using one-way ANOVA followed by Tukey’s post hoc multiple comparison test. As compared with Sham (**a**), MIA (**b**), MIA+Blank (**c**) MIA+ACF nanoemulgel (F10NEG1) and **d** MIA+ACF/Collagen combination (F10CNEG1) treated groups; p < 0.05. Abbreviation: ACF: Aceclofenac nanoemulgel, COMP: complex oligomeric matrix protein, CTXII: C-terminal cross-linked telopeptide of type II collagen, MIA: Mono-iodoacetate

As illustrated in Fig. 6, MIA injection in rats resulted in significantly higher (A) crosslinked C-telopeptides of type II collagen (CTX-II), and (B) Cartilage oligomeric matrix protein (COMP) levels (126.13%, and 575.67%, respectively) in serum as compared to sham group. On the treatment side, a reverse response was observed in rats post treated with ACF nanoemulgel (F10NEG1) and the ACF/collagen combination (F10CNEG1) as compared to the MIA untreated rats, whereas ACF NE reduced (A) CTX-II and (B) COMP levels to 22.22%, and 47.00%, respectively. While the ACF/Collagen combination rectified (A) CTX-II, and (B) COMP to, 42.22% and 72.00%, respectively, when compared to the OA rats. The ACF/Collagen combination showed the best comparable ameliorating effect on these parameters and reached normal values in the healthy control group.

##### Impact of ACF nanoemulgel alone (F10NEG1) or in combination with collagen (F10CNEG1) on serum TNF-α and IHC protein expression of NF-κB-p65in MIA-provoked osteoarthritis.

Serum levels of (A) tumor necrosis factor-alpha (TNF-α), and protein expression of (B) nuclear factor kappa- B (NF-κBp65), were increased 4.13, 15.45 folds in OA rats with regards to that in healthy sham one (p < 0.05). There was no significant difference in the serum level of TNF-α between the blank treated group and MIA OA untreated rats. ACF nanoemulgel (F10NEG1) and ACF/collagen (F10CNEG1) combination treatments reduced the serum TNF-α by 43.59, 66.67%, respectively, as compared to MIA untreated rats (p < 0.05).

**Fig. 7:**
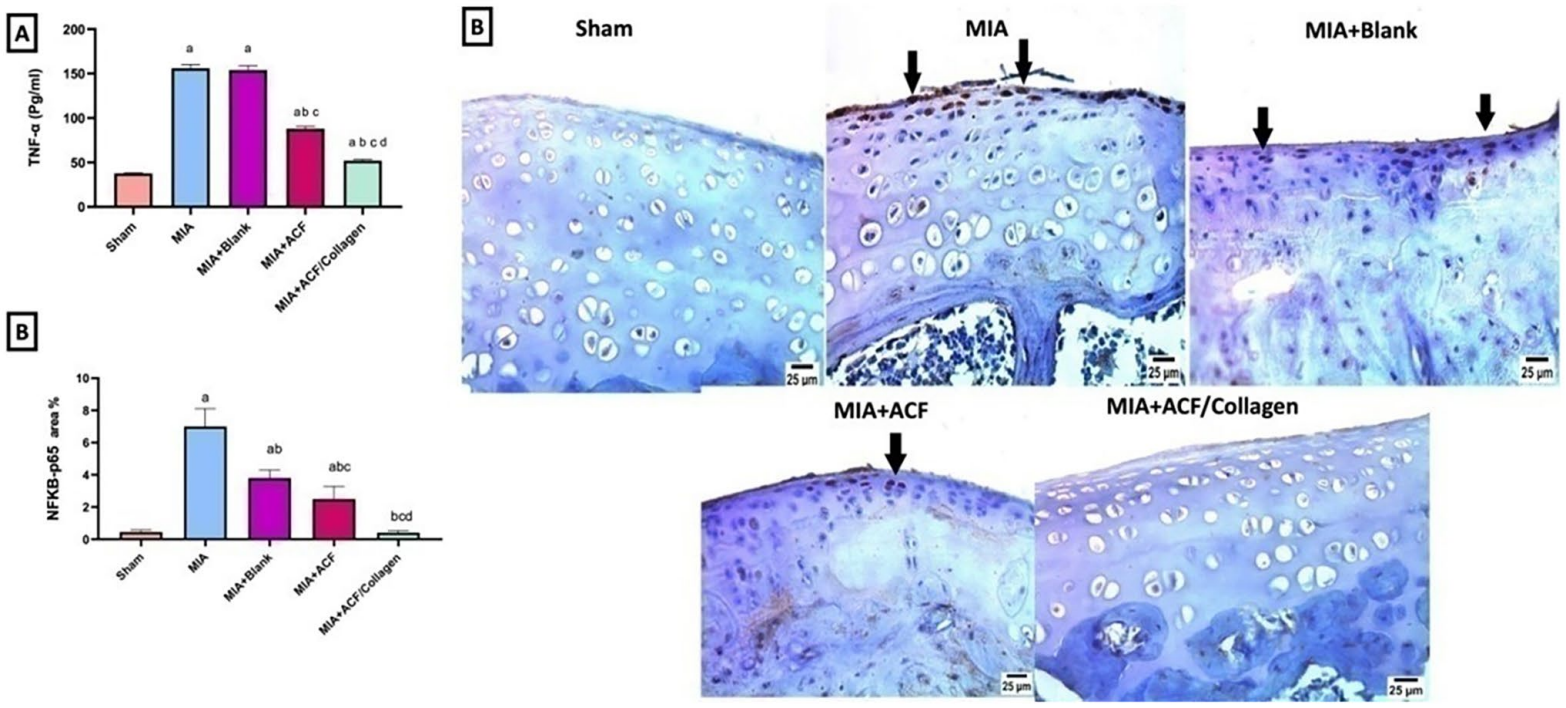
Impact of ACF nanoemulgel alone (F10NEG1) or in combination with collagen (F10CNEG1) on serum levels of **A** TNF-α and IHC protein expression of **B** NF-κB p65in MIA-provoked osteoarthritis. Values are presented as mean (n = 6) ± SD and statistical analysis was carried out using one-way ANOVA followed by Tukey’s post hoc multiple comparison test. As compared with Sham (**a**), MIA (**b**), MIA+Blank (**c**) MIA+ACF and **d** MIA+ACF/Collagen combination (F10CNEG1) treated groups; p < 0.05

Moreover, the OA rats treated with the ACF/collagen combination (F10CNEG1) showed a non-significant difference regarding NF-κBp65compared to the sham group reflecting high degree of protection. Notably, NF-κBp65, protein expression was reduced with the post treatment with the blank (Citronellol oil based nanoemulgel) for fourteen day and that is only positive effect of blank reported in this study, as shown in Fig. 7.

##### Impact of ACF nanoemulgel alone (F10NEG1) or in combination with collagen (F10CNEG1) on knee joint protein expressions of HMGB, RAGE and Klotho in MIA-provoked osteoarthritis

**Fig. 8:**
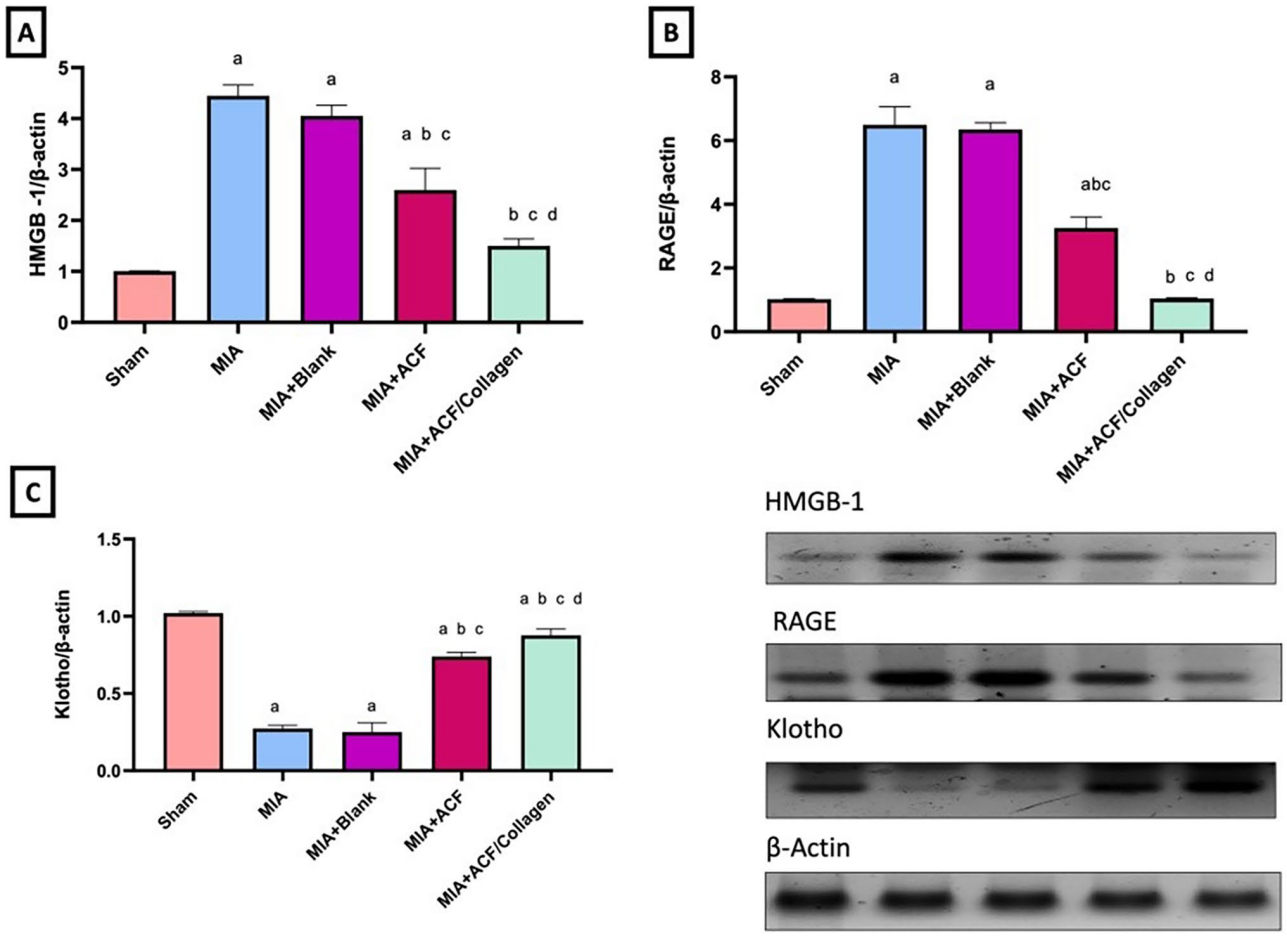
Impact of ACF nanoemulgel alone (F10NEG1) or in combination with collagen (F10CNEG1) on knee joint protein expression of **A** HMGB-1, **B** RAGE and **C** klotho in MIA-provoked osteoarthritis. Values are presented as mean (n = 6) ± SD and statistical analysis was conducted using one-way ANOVA followed by Tukey’s post hoc multiple comparison test. As compared with Sham (**a**), MIA (**b**), MIA+Blank (**c**) MIA+ACF and **d** ACF/Collagen combination (F10CNEG1) treated groups.; p < 0.05. Abbreviation: ACF: Aceclofenac nanoemulgel, HMGB-1: High Mobility Group Box-1, MIA: Mono-Iodoacetate, RAGE: Receptor for Advanced Glycation End products

As presented in Fig. 8, rats with OA experienced higher expression of (A) HMGB-1 and (B) RAGE, with remarkable low (C) klotho expression in the knee joint tissues compared to the sham group (p < 0.05). However, the post treatment with ACF nanoemulgel alone (F10NEG1) or in optimized combined formula(F10CNEG1) significantly decreased (A) HMGB-1 and (B) RAGE as relevant to induction group. Regarding (C) Klotho, both ACF nanoemulgel alone (F10NEG1) and optimized combined formula (F10CNEG1) increased its expression offering protection against articular cartilage damage (p < 0.05). Still the combined formula has the best values as compared to solo treated group and reached normal values of sham group in both HMGB and RAGE parameters.

##### Impact of ACF nanoemulgel alone (F10NEG1) or in combination with collagen (F10CNEG1) on knee joint gene expression of miR-499-5p

**Fig. 9:**
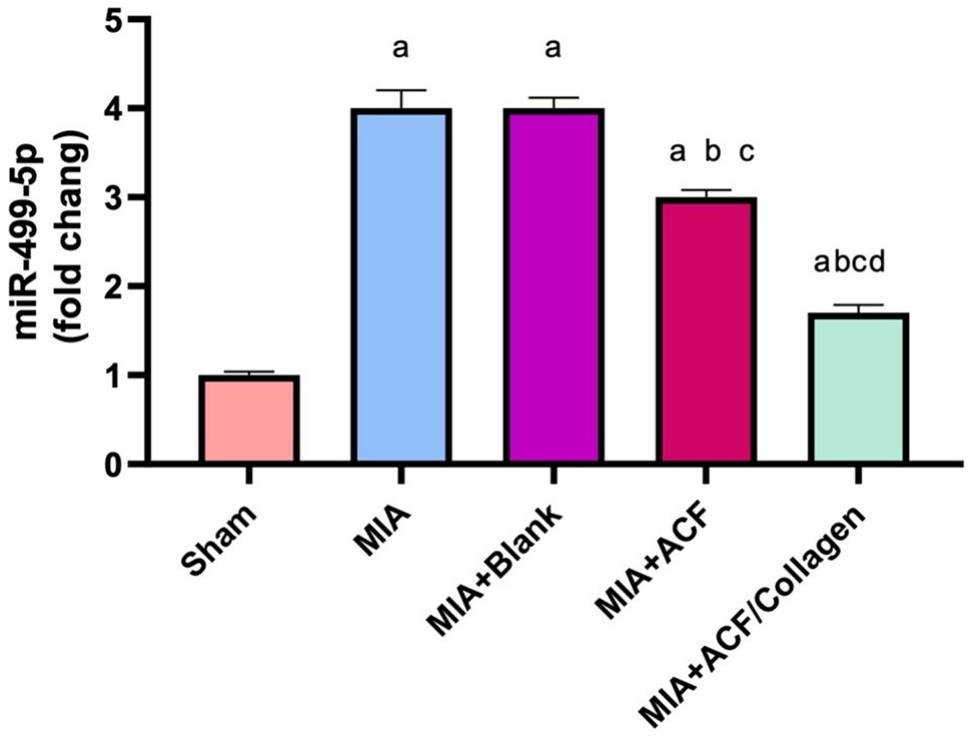
Impact of ACF nanoemulgel alone (F10NEG1) or in combination with collagen (F10CNEG1) on knee joint gene expression of miR-499–5p. Values are presented as mean (n = 6) ± SD and statistical analysis was conducted using one-way ANOVA followed by Tukey’s post hoc multiple comparison test. As compared with Sham (**a**), MIA (**b**), MIA + Blank (**c**) and MIA + ACF(**d**) ACF/Collagen combination (F10CNEG1) treated groups.; p < 0.05. Abbreviation: ACF: Aceclofenac nanoemulgel, MIA: Mono-Iodoacetate, miR-499–5p: MicroRNA -499–5p

In comparison to the sham, the OA rats exhibited fourfold increase in the expression of miR-499-5p (Fig. 9). No significant difference was observed regarding the miR-499-5P expression in the blank treated group and OA untreated rats. Nevertheless, ACF nanoemulgel alone (F10NEG1) or in combination with collagen (F10CNEG1) post treatment after MIA intra-articular injection resulted in a significant decrease in the expression of miR-499-5p as compared to MIA only treated group (p < 0.05). Notably, the combination of ACF and collagen (F10CNEG1) had a more pronounced impact on miR-499-5p than other solo treated groups (p < 0.05).

##### Impact of ACF nanoemulgel alone (F10NEG1) or in combination with collagen (F10CNEG1) on histopathological alterations using H & E stain

**Fig. 10:**
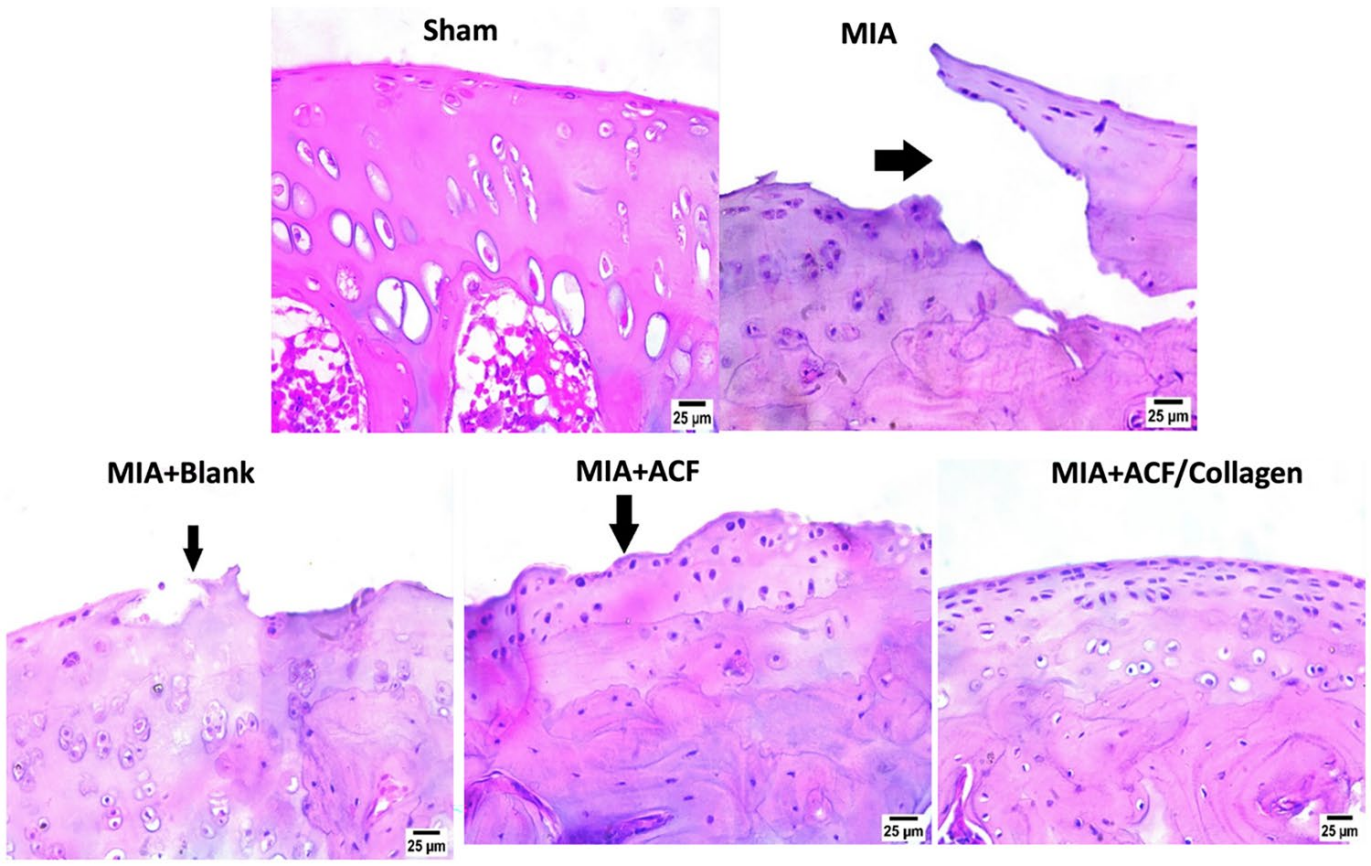
Photomicrographs of cartilage tissue specimens stained by H & E (×40). Abbreviation: ACF: Aceclofenac nanoemulgel, MIA: Mono-Iodoacetate

The arthritic group/MIA group presented sever destruction in articular surface (arrow) as compared to sham group that displayed normal appearance of articular surface and normal histological structure after H & E staining. Similarly, a persistence of osteoarthritis and sever roughness of articular surface (arrow) in the blank treated group. While the ACF treated group showed mild enhancement, but articular surface preference was still rough (arrow). However, arthritic rats that post treated with ACF/collagen combination (F10CNEG1) showed normal histological structure of articular surface (Fig. 10).

##### Impact of ACF nanoemulgel alone (F10NEG1) or in combination with collagen (F10CNEG1) on histopathological alterations using safranin O stain

**Fig. 11:**
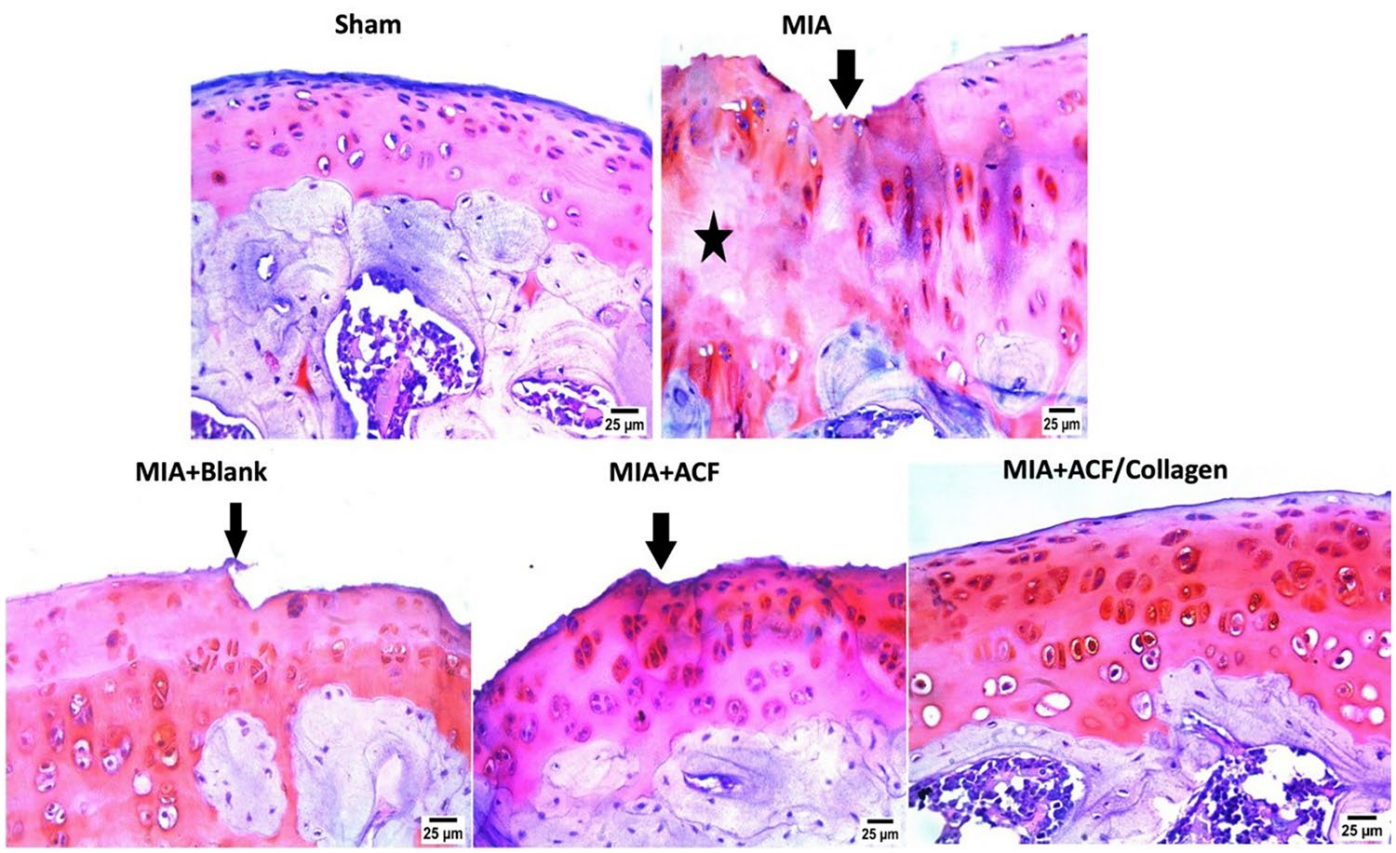
Photomicrographs of cartilage tissue specimens stained by Safranin O stain (×40). Abbreviation: ACF: Aceclofenac nanoemulgel, MIA: Mono-Iodoacetate

Photomicrographs captured after safranin O staining of the cartilage tissue of arthritic rats and blank treated group showed roughness of articular surface(arrow)with partial loss of articular surface comparing to articular surface and histological structure of sham group. Following ACF/collagen combination therapy, a normal histology preference for the articular surface was achieved. Concerning the ACF group marked improvement but still roughness of articular surface persistence (arrow) (Fig. 11).

From the aforementioned results it was clear that in the MIA untreated rats' cartilage the HMGB-1, RAGE and NF-κB p65 expression was significantly increased as compared to the shamgroup. Also, it was observed that there was a significant increase in the serum levels of TNF-α, CTXII, and COMP. This was inline with previous studythat illustrated that HMGB-1 level was increased in cartilage, synovium and synovial fluid of OA patients which was found to be inversely associated with disease severity [69]. Another study confirmed our results, as it showed RAGE expressions were markedly elevated in MIA-inducing OA in ratsand considerably decreased by CoQ10 therapy [70]. According to previous report, dysregulated NF-κB pathway activity, which is linked to increased inflammatory cytokine production, is a significant factor in MIA-induced OA in rats [71]. It was also illustrated preiously in more than one study that MIA that was used for induction of OA could raise both COMP and CTX-II levels, which were linked to the development of OA [72, 73].

We may draw conclusions based on our findings and the findings of others that HMGB-1, which is a nuclear protein that could be secreted by chondrocytes to the extracellular matrix in the latter stages of OA, encourage a catabolic transition in chondrocytes. This would be associated with elevation in the serum levels of COMP and CTX-II as indicator of damage in articular cartilage in OA model [74]. Moreover, HMGB-1 could bind to RAGE cell surface receptor, subsquently activate NF-κB, the known transcription factor, increasing the production of inflammatory cytokines such as TNF-αand this mechanism was confirmed previously in rat temporomandibular joint osteoarthritis (TMJOA) induced by MIA [75] Another invitro study [76], showed that extracellular HMGB-1 can promote RAGE expression in a variety of cell types, which was consistent with our work findings.

Our results illustrated that OA-untreated animals showed considerably lower Klotho cartilage expression as compared to sham rats. This was confirmed by previous studies where Klotho expression in cartilages was down- regulated in OA mice model [77] and rat model [43]. Furthermore, as compared to the shamed rat, the expression level of miR-499-5p, or currently referred as miR-499a was considerably higher in the MIA-induced OA animals. Our results were previously confirmed, as it was shown that MiR-499a levels were elevated in OA human cartilage, especially in late-stage OA. It was explained that by suppressing growth differentiation factor-5 (GDF5), miR-499a promoted chondrocyte extracellular matrix breakdownand increased levels of CTXII and COMP [78]. Also, another in-vivostudy of OA rat model confirmed the role of miR-499a inhibition, in boosting cartilage repair and avoiding disease progression [79].

Citronellol is a form of monoterpene alcohol present in many essential oils of plants that has a variety of pharmacological actions including antifungal, antibacterial, anticonvulsant, and analgesic properties [4]. Dose dependent anti-inflammatory effect of citronellol had been reported when administered (50, 100, 200 mg/kg,IP) in Carrageenan- and arachidonic acid-induced hind paw edema in rats [3]. Another study reported that citronellol possessed anti-nociceptive and anti-inflammatory effects through its assessement in acetic-acid-induced abdominal writhing, formalin-induced licking, thermal model of pain and carrageenan-induced pleurisy in mice. Moreover, citronellol was capable in invitro study to reduce the nitric oxide production by LPS-stimulated macrophage [80]. Furthermore, citronellol at a dose of 50 mg/kg BW administered orally to DMBA-treated rats dramatically reduced the expression of NF-κB and inflammatory parameters in mammary tissues [81].

Nonetheless, all the aforementioned reported effects knee joints of OA rats treated with nanoemulgel citronellol (blank) displayed sever destruction and roughness of articular surface similar to MIA treated group findings. On the biochemical level, values from animals post-treated with blank after MIA-provoked osteoarthritis showed no appreciable positive change and did not significantly differ from the MIA solo treated group except for NF-κBp65 findings, where a significant reduction was observed in the blank treated group relevant to the induction untreated group. However, this reduction was not enough and did not prevent MIA deleterious effect on knee joint in blank treated group. The ACF citronellol nanoemulgel and collagen integrated ACF citronellol nanoemulgel were capable of drastically lowering HMGB-1, RAGE, and NF-κB p65 cartilage expression in MIA rats, as well as serum levels of TNF-α, CTXII, and COMP with the most significant reduction offered by the collagen integrated ACF citronellol nanoemulgel. This augmented effect is suggestibly resulted from the previously mentioned observed effect on the blank treated group of citronellol on reduction of NF-κBp65. Moreover, there was significant elevation in the expression level of Klotho in the cartilages of both treated groups with ACF citronellol nanoemulgel and collagen integrated ACF citronellol nanoemulgel. Also, in both aforementioned treated groups there was significant mitigation in the expression level of miR-499-5p.

ACF is a known COX-2 inhibitor that possess analgesic and anti-inflammatory effects via decreasing PGE2 production directly through inhibition COX-2 enzyme and indirectly through mitigating the COX-2 synthesis in the cartilage [82]. Additionally, studies have shown that ACF may promote the manufacture of glycosaminoglycansan important component of healthy cartilage [83]. Past study reported that aceclofenc could modulate NF-κB pathway and attenuated experimental RA [84]. Additionally, and in line with our results ACF significantly inhibited LPS-stimulated osteoarthritic synovial membranes IL-1 and TNF-α synthesis [85].

As further documentation of the effectiveness of our prepared formula, histopathological investigations were excluded using H & E as well as safranin O staining. Totally, OA untreated rats and blank treated group showed severe deterioration of the articular surface compared and degenerated chondrocytes to the sham group, these histological findings were reported previously [39, 86]. While in the ACF-treated group there was little improvement but in the ACF/collagen combination treated group, prevented MIA destructive effect on knee joint and confirmed regeneration and chondroprotection.

## Conclusion

Nanoemulgel is a recently developed nanofrmulation with promising outcomes as a drug delivery system for topically applied drugs. It showed higher stability than nanoemulsions, more patient satisfaction since it is non-oily in nature, and better spreadability [54]. A new nanoemulgel formula which contains aceclofenac, citronellol oil, and collagen was successfully developed. This new formula has the advantage of increasing aceclofenac permeability through the skin while benefiting from the combined components synergistic anti-inflammatory impact of both aceclofenac and citronellol in the treatment of osteoarthritis. This study can infer that collagen based ACF/citronellol nanoemulgel were able to modulate HMGB-1/RAGE/NF-κB pathway, mitigating the production of inflammatory cytokine TNF-α. They were also able to modulate Klotho andmiR-499, reducing serum CTXII and COMP, by reducing the cartilage destruction. Hence, the findings of the current work encourage the use of this promising combined formula in treatment of OA pateints.

## Data Availability

The datasets generated during and/or analyzed during the current study are available from the corresponding author on reasonable request.
